# Association of Work Schedules With Nurse Turnover: A Cross-Sectional National Study

**DOI:** 10.3389/ijph.2023.1605732

**Published:** 2023-04-24

**Authors:** Sung-Heui Bae

**Affiliations:** College of Nursing, Graduate Program in System Health Science and Engineering, Ewha Womans University, Seoul, Republic of Korea

**Keywords:** earning, job satisfaction, nurse turnover, overtime, work hours

## Abstract

**Objective:** To examine the relationship of work schedules with nurse turnover across various work settings.

**Methods:** A cross-sectional study design was used with data collected from 17,046 nurses who participated in the 2018 National Sample Survey of Registered Nurses in the U.S. Multivariate logistic regression was used to examine the effects of work hours and overtime on nurse turnover.

**Results:** Longer weekly work hours increased nurse turnover (OR = 1.104, 95% confidence interval [CI] = 1.006–1.023). A non-linear relationship was observed between overtime and turnover. Compared with nurses with no overtime, the turnover for nurses working 1–11 h overtime per week decreased (OR = 0.893, 95% CI = 0.798–0.999). When nurses worked ≥12 h, turnover increased (OR = 1.260, 95% CI = 1.028–1.545). Earning from the primary nursing position decreased turnover among nurses working in hospitals, other inpatient settings, and clinics. Job satisfaction decreased turnover.

**Conclusion:** To prevent nurse turnover, it is important to monitor and regulate nurses’ working hours at institutional and government levels. Government support and policy implementations can help prevent turnover.

## Introduction

Nursing shortage is a critical issue in many countries (1); nursing turnover produces and aggravates this shortage (2). High turnover and low retention of qualified nurses have great impacts on various aspects of healthcare, increasing the pressure to provide high quality, cost-effective nursing services (3). Turnover is costly both economically and non-economically (4). Due to turnover, healthcare organizations can lose intellectual capital and experience productivity losses (5). In the U.S., the annual turnover rate for nurses is 27.65% (6), while it is 13.3% in South Korea (7).

When healthcare organizations experience high levels of nurse turnover, they must bear the high costs of hiring and training new nurses (4). Ruiz et al. (8) reported that the total cost of turnover was three times the average nursing staff salary. With a high workload resulting from a shortage of staff, burnout can lead to the turnover of the remaining nurses and create a shortage-turnover cycle (9). High turnover of nurses can affect their mental health and job satisfaction, as well as patient outcomes by creating unhealthy work environments (10–12).

Working conditions have been reported to be the most critical factor for nurse retention (13). A recent systematic review (14) found multifaceted determinants of turnover at the individual and organizational levels. The determinants included stress, job dissatisfaction, managerial style, and supervisory support, all of which were related to work conditions. Among the work conditions, work schedules can affect nurse turnover. Given the shortage, nurses might need to work longer hours that can increase their intent to leave, as indicated by Stimpfel et al. (15), who found that longer shift length increased such intent. In contrast, in other studies, when overtime or voluntary overtime hours increased, nurse turnover decreased (7, 16). The relationship between nurses’ work schedules and turnover has not been examined extensively. Further, the characteristics of work schedules that could lead to an increase in nurse turnover are yet to be understood.

Nurses work in various settings, with different schedules. Their work schedules can be measured by shift length, work hours per week, overtime, and breaks between and within shifts (17). Because hospital nurses provide continuous care, they often work long hours without sufficient breaks and return to work quickly, which can affect their fatigue (18). Such work schedules negatively affect the recovery time and vigilance levels, which in turn, negatively affect patient outcomes. A recent systematic review found that working more than 12 h a day or more than 40 h per week was related to adverse patient outcomes (17). To prevent such adverse schedules, several states in the U.S. have regulated nurses’ working hours (e.g., not working more than 12 h within 24 h) and banned mandatory overtime (19).

Furthermore, nurse turnover has often been examined in hospital settings (14). A few studies have focused on this in nursing homes (20, 21). Most of the nurses in the U.S. worked in a hospital, while the remaining worked in clinics/ambulatory and other inpatient settings (22). However, nurse turnovers in settings other than hospitals have not been adequately examined. As turnover could differ based on the work setting, the relationship between work schedule and turnover can be different in each setting. Understanding nurse turnover and its relationship with work schedules in various work settings is important. Exploring this relationship can provide practical benefits and instructions for management practice regarding the work schedules of nurses. Institutional policies developed based on this study’s findings can contribute to reduce nurse turnover.

The Brewer-Kovner synthesis model of direct turnover influences (16) was used to guide this study. This model explains the impact of work attributes on turnover along with personal characteristics, opportunity, work attitudes, and shocks (e.g., injuries). Considering that work schedule is considered one of the work attributes, this study aimed to examine nurse turnover and its relationship with work schedules in various work settings using the data from the 2018 National Sample Survey of Registered Nurses (NSSRN). Using the NSSRN, this study’s findings can present the current status of nurse turnover and provide evidence for policy implementation regarding nurses’ work schedules.

## Methods

### Study Design

A cross-sectional study design was adopted to investigate the impact of work schedule on nurse turnover across various work settings using data from the 2018 NSSRN (22). The 2018 NSSRN data were collected from 50,273 active registered nurses licensed from all U.S. states between April and October 2018. These data are the most recent and are publicly available. The review and consent exemption for this study was approved from the university’s Institutional Review Board because of the use of publicly available de-identified data.

### Sample and Data Collection

The sampling frame of the 2018 NSSRN was based on a list of registered nurses (RNs) from the National Council of State Board of Nursing and individual State Boards of Nursing. Stratified sampling was conducted based on RNs who possessed a nurse practitioner (NP) license and those who did not. A survey questionnaire was sent to 52,255 RNs and 50,265 NPs. The number of responses was 50,273, with a 49.0% national weighted response rate. The present study included the data for RNs who were employed on 31 December 2017, worked as RN and not as NP. The inclusion criteria for this study were that nurses should be a) licensed RNs and b) working full time. Thus, data for 17,060 RNs were included, and after excluding the data with missing study variables, data from 17,046 RNs were used. With an odds ratio (OR) of 1.2, power of 0.95, and significance level of 0.05, the sample size was calculated as 2,451 according to the logistic regression analysis conducted using G-Power 3.1.9.4 (23). The sample size in this study was 17, 046, which was higher than the target sample size.

### Measures

The 2018 NSSRN survey evaluated eligibility and education, primary nursing employment details, whether left or remained in primary nursing position, presence of secondary employment, whether recognized as a nurse practitioner, whether working in fields other than nursing, presence of prior nursing employment, opinions on national practitioner data bank, license and certification details, and general information.

Nurse turnover was measured by asking nurses if they left their primary nursing position, in which they remained until 31 December 2017. Since the data collection was conducted between April and October 2018, nurse turnover was examined based on nurses leaving their job from 31 December 2017 until the data collection time.

Work schedule characteristics for the primary nursing position nurses held on 31 December 2017, were measured by weekly scheduled and actual work hours. The difference between them was used to calculate overtime hours per week. For the analytical models, actual weekly work hours and overtime hours per week were used. Furthermore, considering a 12-h shift, the categorical variable of overtime hours per week (0, 1–11, and ≥12) was also used for the analytical model. If nurses worked over 12 h a week, they might work one additional 12-h shift or more per week.

In addition to work schedule, the Brewer-Kovner synthesis model of turnover (16) includes other variables that affect turnover. Among them, this study included several individual and work-related characteristics. Specifically, the individual characteristics included were sex, age in 2018, race/ethnicity, highest nursing education, marital status, having a dependent (<6 years old at home), and pre-tax annual total household income in 2017. Work-related characteristics of the primary nursing position nurses held on 31 December 2017 included work setting, pre-tax annual earning, percentage of patient care time, unionization, whether engaged in any other position, and job satisfaction. Work settings were categorized into hospital (e.g., inpatient unit), other inpatient settings (e.g., nursing home unit, inpatient mental health), clinic/ambulatory (e.g., nurse-managed health center), and other types of settings (e.g., home health agencies). The supplementary table presents details of the work settings. Job satisfaction was measured using one item (“How satisfied were you with the primary nursing position you held on 31 December 2017?”) on a four-point Likert scale.

### Statistical Analysis

SAS version 9.4 was used for data analyses. Descriptive statistics, chi-square tests, and t-tests were used for the statistical analyses. As mentioned above, overtime hours per week was used as continuous and categorical variables (0, 1–11, and ≥12). The impact of work schedules on nurse turnover was analyzed using multivariate logistic regression analyses. To examine this impact in each work setting, participants’ data were analyzed using work settings. Additionally, two types of overtime hour variables were used in each work setting model.

## Results

### Participant Characteristics

The characteristics of 17,046 nurses are presented in Table 1. A total of 2,250 nurses (13.2%) left their primary nursing position. On average, nurses worked 41 h per week, and approximately 30% worked more than 40 h per week. Among the nurses, 43% reported that they worked overtime, which means working outside of scheduled work hours. A total of 1,304 (7.6%) nurses worked 12 or more hours of overtime per week. The nurses’ mean age was 47.73 ± 12.31 years, with 70% being married. Regarding work settings, more than half (58%) worked in hospitals and 9% worked in other inpatient settings, such as nursing homes. Approximately 33% of the participants worked in either clinic/ambulatory or other types of settings. Most nurses were satisfied with their primary nursing positions, while 10.7% reported dissatisfaction with their positions.

**TABLE 1:** General characteristics of study variables (N = 17,046) (United States, 2023).

Variables	Total		Turnover (Yes)		Turnover (No)		*p*-value
	N (%)	Mean (SD)	N (%)	Mean (SD)	N (%)	Mean (SD)	
Total	17,046 (100.0)		2,250 (13.2)		14,796 (86.8)		
Work schedule characteristics							
Weekly scheduled work hours		38.24 (5.19)		38.42 (5.48)		38.22 (5.14)	0.078
Weekly actual work hours		41.32 (7.68)		42.37 (8.76)		41.16 (7.49)	<0.001
40 or less	11,682 (68.5)		1,453 (12.4)		10,229 (87.6)		<0.001
41–59	4,623 (27.1)		644 (13.9)		3,979 (86.1)		
60 or more	741 (4.4)		153 (20.7)		588 (79.3)		
Overtime hours per week		3.31 (5.48)		4.18 (6.34)		3.18 (5.32)	<0.001
0	9,658 (56.7)		1,172 (12.1)		8,486 (87.9)		<0.001
1–11	6,084 (35.7)		809 (13.3)		5,275 (86.7)		
12 or more	1,304 (7.6)		269 (20.6)		1,035 (79.4)		
Overtime hours (only >0)		7.64 (6.01)		8.72 (6.64)		7.45 (5.88)	<0.001
Nurse characteristics							
Sex							
Male	1,682 (9.9)		253 (15.0)		1,429 (85.0)		0.019
Female	15,364 (90.1)		1,997 (13.0)		13,367 (87.0)		
Age, years in 2018		47.73 (12.31)		46.26 (13.47)		47.95 (12.11)	<0.001
Race/ethnicity							
Non-Hispanic White	13,593 (79.7)		1,796 (13.2)		11,797 (86.8)		0.920
Other	3,453 (20.3)		454 (13.2)		2,999 (86.8)		
Highest nursing education							
Diploma degree	820 (4.8)		98 (12.0)		722 (88.0)		0.247
Associate degree	5,119 (30.0)		717 (14.0)		4,402 (86.0)		
Bachelor’s degree	7,848 (46.1)		1,011 (12.9)		6,837 (87.1)		
Master’s degree	2,900 (17.0)		382 (13.2)		2,518 (86.8)		
PhD/DNP in nursing	359 (2.1)		42 (11.7)		317 (88.3)		
Marital status							
Married or in domestic partnership	11,943 (70.1)		1,479 (12.4)		10,464 (87.6)		<0.001
Widowed, divorced, separated	2,825 (16.6)		388 (13.7)		2,437 (86.3)		
Never married	2,278 (13.3)		383 (16.8)		1,895 (83.2)		
Dependent (less than 6 years old at home)							
Yes	2,543 (14.9)		354 (13.9)		2,189 (86.1)		0.244
No	14,503 (85.1)		1,896 (13.1)		12,607 (86.9)		
Household income ($)							
$50,000 or less	706 (4.2)		152 (21.5)		554 (78.5)		<0.001
$50,001 to $75,000	3,156 (18.5)		512 (16.2)		2,644 (83.8)		
$75,001to $100,000	4,097 (24.0)		528 (12.9)		3,569 (87.1)		
$100,001 to $150,000	5,341 (31.3)		636 (11.9)		4,705 (88.1)		
More than $150,000	3,746 (22.0)		422 (11.3)		3,324 (88.7)		
Work-related characteristics							
Work settings							
Hospital	9,980 (58.5)		1,347 (13.5)		8,633 (86.5)		<0.001
Other inpatient setting	1,525 (9.0)		289 (19.0)		1,236 (81.1)		
Clinic/ambulatory	2,359 (13.8)		236 (10.0)		2,123 (90.0)		
Other types of setting	3,182 (18.7)		378 (11.9)		2,804 (88.1)		
Earning ($1,000)		77.36 (31.34)		72.38 (30.19)		78.12 (31.44)	<0.001
Patient care time (%)		51.37 (34.19)		52.80 (33.20)		51.15 (34.33)	0.033
Unionization							
Yes	2,673 (15.7)		291 (10.9)		2,382 (89.1)		<0.001
No	14,373 (84.3)		1,959 (13.6)		12,414 (86.4)		
Held any other positions							
Yes	1,761 (10.3)		247 (14.0)		1,514 (86.0)		0.279
No	15,285 (89.7)		2,003 (13.1)		13,282 (86.9)		
Job satisfaction		3.28 (0.72)		2.87 (0.90)		3.35 (0.66)	<0.001
Extremely dissatisfied (1)	411 (2.4)		226 (55.0)		189 (45.0)		<0.001
Moderately dissatisfied (2)	1,409 (8.3)		430 (30.5)		979 (69.5)		
Moderately satisfied (3)	8,225 (48.2)		1,056 (12.8)		7,169 (87.2)		
Extremely satisfied (4)	7,001 (41.1)		538 (7.7)		6,463 (92.3)		

Note. SD, standard deviation; PhD, Doctor of Philosophy; DNP, Doctor of Nursing Practice.

### Multivariate Logistic Regression

Logistic regression was used to examine the impact of actual work schedule on turnover among nurses (Table 2). The models using different work settings are presented in Tables 3, 4, which examined the work-setting-specific impacts of work schedules on turnover. Model 1 was the total model, including actual weekly work hours and overtime hours per week. Longer weekly work hours increased nurse turnover (OR = 1.104, 95% confidence interval [CI] = 1.006–1.023). For Model 2, which used categorical variables of overtime, the major difference was the emergence of overtime hours per week. Compared to the turnover for nurses who did not work overtime, that for RNs who worked 1–11 h of overtime decreased (OR = 0.893, 95% CI = 0.798–0.999). When they worked ≥12 overtime hours per week, turnover increased (OR = 1.260, 95% CI = 1.028–1.545). In terms of work settings, compared with RNs who worked in hospitals, those who worked in other inpatient settings showed increased turnover. Nurse turnover decreased in clinic/ambulatory and other types of settings. In both models, sex, age, household income, nurses’ earnings from their primary position, unionization, and job satisfaction were significantly associated with nurse turnover.

**TABLE 2:** Work schedules contributing to nurse turnover (N = 17,046) (United States, 2023).

Variables	Model 1			Model 2			
	OR	95% CI		OR		95% CI	
Work schedule characteristics							
Weekly actual work hours	1.014**	1.006–1.023		1.017**		1.010–1.025	
Overtime hours per week (ref: 0)	1.012	1.000–1.023					
1–11				0.893*		0.798–0.999	
12 or more				1.260*		1.028–1.545	
Nurse characteristics							
Sex (ref: female)							
Male	1.170*	1.007–1.360		1.164*		1.001–1.353	
Age, years in 2018	0.995*	0.990–0.999		0.995*		0.990–1.000	
Race/ethnicity (ref: non-Hispanic White)							
Other	0.964	0.858–1.084		0.952		0.847–1.071	
Highest nursing education (ref: diploma degree)							
Associate degree	0.969	0.765–1.228		0.965		0.762–1.223	
Bachelor’s degree	0.925	0.731–1.171		0.923		0.729–1.168	
Master’s degree or PhD/DNP in nursing	1.136	0.885–1.458		1.139		0.887–1.462	
Marital status (ref: Married or in domestic partnership)							
Widowed, divorced, separated	1.034	0.902–1.185		1.033		0.902–1.184	
Never married	1.133	0.976–1.315		1.138		0.981–1.321	
Dependent (less than 6 years old at home, ref: yes)							
No	1.010	0.877–1.164		1.009		0.876–1.163	
Household income (ref: $50,000 or less, $)							
$50,001 to $75,000	0.775*	0.625–0.962		0.775*		0.624–0.961	
$75,001 to $100,000	0.686**	0.548–0.857		0.685**		0.548–0.856	
$100,001 to $150,000	0.691**	0.549–0.870		0.690**		0.548–0.869	
More than $150,000	0.731*	0.567–0.943		0.733*		0.568–0.945	
Work-related characteristics							
Work setting (ref: hospital)							
Other inpatient setting	1.240**	1.065–1.444		1.238**		1.063–1.442	
Clinic/ambulatory	0.753**	0.646–0.878		0.753**		0.645–0.878	
Other types of setting	0.865*	0.755–0.990		0.855*		0.746–0.980	
Earning ($1,000)	0.994**	0.992–0.997		0.994**		0.992–0.996	
Patient care time (%)	1.001	1.000–1.003		1.001		1.000–1.003	
Unionization (ref: yes)							
No	1.237**	1.077–1.420		1.246**		1.085–1.431	
Held any other positions (ref: yes)							
No	0.916	0.789–1.065		0.924		0.795–1.074	
Job satisfaction	0.441**	0.415–0.468		0.439**		0.413–0.466	

Note. OR, odds ratio; CI, confidence interval, PhD (Doctor of Philosophy); DNP (Doctor of Nursing Practice) *p < 0.05, **p < 0.01.

**TABLE 3:** Work schedules contributing to nurse turnover in hospitals and other inpatient settings (United States, 2023).

Setting	Hospitals				Other inpatient setting			
	Model 3		Model 4		Model 5		Model 6	
Variables	OR	95% CI	OR	95% CI	OR	95% CI	OR	95% CI
Work schedule characteristics								
Weekly actual work hours	1.022**	1.011–1.034	1.022**	1.012–1.033	1.013	0.990–1.037	1.026*	1.005–1.047
Overtime hours per week (ref:0)	0.999	0.982–1.015			1.041*	1.008–1.074		
1–11			0.863*	0.749–0.995			0.884	0.625–1.249
12 or more			1.113	0.851–1.456			1.612	0.933–2.784
Nurse characteristics								
Sex (ref: female)								
Male	1.264*	1.058–1.511	1.256*	1.050–1.501	0.796	0.490–1.293	0.806	0.496–1.308
Age, years in 2018	0.993*	0.987–0.999	0.993*	0.987–0.999	0.996	0.982–1.010	0.995	0.981–1.009
Race/ethnicity (ref: non-Hispanic White)								
Other	0.886	0.762–1.030	0.875	0.752–1.018	1.132	0.807–1.589	1.116	0.795–1.566
Highest nursing education (ref: diploma degree)								
Associate degree	1.056	0.750–1.486	1.057	0.750–1.488	0.805	0.427–1.519	0.816	0.433–1.538
Bachelor’s degree	1.107	0.789–1.552	1.111	0.792–1.559	0.631	0.327–1.216	0.627	0.326–1.207
Master’s degree or PhD/DNP in nursing	1.549*	1.082–2.218	1.558*	1.088–2.232	0.811	0.390–1.685	0.826	0.397–1.715
Marital status (ref: Married or in domestic partnership)								
Widowed, divorced, separated	1.106	0.922–1.328	1.104	0.920–1.325	1.294	0.896–1.868	1.281	0.887–1.850
Never married	1.199*	1.002–1.435	1.205*	1.007–1.443	1.042	0.645–1.683	1.009	0.624–1.630
Dependent (less than 6 years old at home, ref: yes)								
No	0.909	0.760–1.086	0.906	0.757–1.083	1.304	0.847–2.008	1.286	0.834–1.983
Household income (ref: $50,000 or less, $)								
$50,001 to $75,000	0.825	0.625–1.088	0.822	0.624–1.085	0.832	0.440–1.575	0.834	0.442–1.577
$75,001 to $100,000	0.699*	0.524–0.934	0.696*	0.521–0.929	0.944	0.483–1.845	0.940	0.482–1.835
$100,001 to $150,000	0.696*	0.516–0.939	0.693*	0.514–0.934	0.905	0.446–1.836	0.894	0.441–1.812
More than $150,000	0.727	0.521–1.014	0.726	0.520–1.012	1.232	0.568–2.671	1.230	0.568–2.664
Work-related characteristics								
Earning ($1,000)	0.994**	0.991–0.996	0.994**	0.991–0.996	0.989**	0.982–0.996	0.989**	0.982–0.996
Patient care time (%)	1.002*	1.000–1.005	1.002*	1.000–1.005	0.999	0.995–1.004	0.999	0.995–1.004
Unionization (ref: yes)								
No	1.135	0.960–1.343	1.141	0.965–1.350	1.219	0.737–2.017	1.277	0.771–2.114
Held any other positions (ref: yes)								
No	0.825	0.681–1.000	0.838	0.691–1.016	0.841	0.534–1.324	0.855	0.542–1.347
Job satisfaction	0.477**	0.440–0.517	0.475**	0.438–0.514	0.357**	0.299–0.427	0.352**	0.295–0.421
N	9,980		9,980		1,525		1,525	

Note. OR, odds ratio; CI, confidence interval, PhD (Doctor of Philosophy); DNP (Doctor of Nursing Practice) *p < 0.05, **p < 0.01.

**TABLE 4:** Work schedules contributing to nurse turnover in clinic/ambulatory and other types of settings (United States, 2023).

Setting	Clinic/ambulatory				Other types			
	Model 7		Model 8		Model 9		Model 10	
Variables	OR	95% CI	OR	95% CI	OR	95% CI	OR	95% CI
Work schedule characteristics								
Weekly actual work hours	1.006	0.978–1.036	1.019	0.992–1.046	0.993	0.972–1.014	0.993	0.974–1.012
Overtime hours per week (ref:0)	1.029	0.995–1.064			1.019	0.994–1.045		
1–11			0.911	0.647–1.282			1.043	0.786–1.385
12 or more			1.447	0.677–3.090			1.610	0.996–2.602
Individual characteristics								
Sex (ref: female)								
Male	1.130	0.595–2.147	1.102	0.581–2.091	1.112	0.727–1.701	1.101	0.719–1.685
Age, years in 2018	1.014	0.998–1.030	1.015	0.999–1.030	1.002	0.990–1.015	1.003	0.990–1.015
Race/ethnicity (ref: non-Hispanic White)								
Other	1.476*	1.028–2.119	1.466*	1.021–2.107	0.917	0.680–1.237	0.911	0.675–1.230
Highest nursing education (ref: diploma degree)								
Associate degree	1.579	0.788–3.165	1.547	0.771–3.103	0.759	0.468–1.232	0.749	0.462–1.215
Bachelor’s degree	1.173	0.581–2.366	1.154	0.571–2.331	0.719	0.444–1.164	0.709	0.438–1.149
Master’s degree or PhD/DNP in nursing	1.149	0.547–2.411	1.145	0.546–2.403	0.774	0.470–1.274	0.763	0.464–1.257
Marital status (ref: Married or in domestic partnership)								
Widowed, divorced, separated	1.029	0.669–1.582	1.036	0.673–1.594	0.772	0.562–1.060	0.771	0.561–1.060
Never married	1.400	0.810–2.418	1.413	0.819–2.438	0.606*	0.377–0.975	0.612*	0.380–0.984
Dependent (less than 6 years old at home, ref: yes)								
No	1.694*	1.102–2.605	1.719*	1.119–2.641	1.161	0.788–1.709	1.167	0.793–1.718
Household income (ref: $50,000 or less, $)								
$50,001 to $75,000	0.746	0.397–1.401	0.745	0.396–1.402	0.561*	0.318–0.990	0.566*	0.321–0.999
$75,001 to $100,000	1.017	0.546–1.895	1.031	0.553–1.922	0.385**	0.215–0.689	0.389**	0.217–0.698
$100,001 to $150,000	0.943	0.501–1.777	0.950	0.504–1.793	0.474*	0.263–0.853	0.482*	0.267–0.870
More than $150,000	0.881	0.437–1.776	0.908	0.450–1.833	0.468*	0.246–0.891	0.472*	0.248–0.899
Work-related characteristics								
Earning ($1,000)	0.992*	0.985–0.998	0.992*	0.985–0.998	1.000	0.995–1.005	1.000	0.995–1.005
Patient care time (%)	0.999	0.994–1.005	0.999	0.994–1.005	1.000	0.996–1.004	1.000	0.996–1.004
Unionization (ref: yes)								
No	1.625*	1.040–2.538	1.622*	1.038–2.545	1.506*	1.009–2.247	1.519*	1.018–2.267
Held any other positions (ref: yes)								
No	0.873	0.565–1.347	0.866	0.560–1.337	1.479	0.991–2.210	1.478	0.990–2.207
Job satisfaction	0.379**	0.316–0.455	0.377**	0.314–0.452	0.402**	0.351–0.461	0.402**	0.351–0.461
N	2,359		2,359		3,182		3,182	

Note. OR, odds ratio; CI, confidence interval, PhD (Doctor of Philosophy); DNP (Doctor of Nursing Practice) *p < 0.05, **p < 0.01.

Models 3 and 4 included nurses working in hospitals. Weekly actual work hours increased nurse turnover (OR = 1.022, 95% CI = 1.011–1.034). Overtime hours per week was not related to nurse turnover. However, in Model 4, using categorical variables of overtime, nurses working 1–11 h of overtime reported lower turnover compared to those not working overtime (OR = 0.863, 95% CI = 0.749–0.995). In both models, higher age, household income, nurses’ earnings, and job satisfaction decreased nurse turnover. Being a male nurse, having a higher degree in nursing, being unmarried, and providing greater percentage of patient care time increased turnover.

Models 5 and 6 analyzed nurses working in other inpatient settings such as nursing homes. For Model 5, actual weekly work hours and overtime hours were used as continuous variables. Only overtime hours per week increased turnover (OR = 1.041, 95% CI = 1.008–1.074). However, in Model 6, using categorical variables of overtime, only weekly actual work hours increased nurse turnover (OR = 1.026, 95% CI = 1.005–1.047). Higher earnings from the primary nursing position and job satisfaction were predictors of a decrease in nurse turnover.

Models 7 to 10 present the logistic regression findings of nurse turnover in clinics/ambulatory and other types of settings. Neither the actual weekly work hours nor overtime hours were significant predictors of nurse turnover. The significant predictors of increased nurse turnover in clinic and ambulatory settings were race/ethnicity, having a dependent, lower earnings from the primary nursing position, and non-unionized workplace. Higher job satisfaction decreased nurse turnover. Being in other types of settings, being unmarried, higher household income, and higher job satisfaction reduced turnover. Working in a non-unionized workplace increased nurse turnover.

## Discussion

This study investigated nurse turnover and its relationship with work schedule across four work settings. To understand the turnover rate, data were collected between April and October 2018, and turnover experience was based on the primary position of the nurses until 31 December 2017. Thus, the average turnover rate was 13.2% across all work settings, with 13.5% only for hospital nurses (Table 1). This is lower than the findings of a previous study (27.65%) (6). Among the four work settings, nurses in other inpatient settings such as nursing homes, long-term care, and inpatient mental health reported the highest turnover rate (19.0%, Table 1). A high rate of nurse turnover has been a critical issue for nursing homes (21), and this study confirmed this issue compared to other settings, implying that more attention needs to be paid to nurses working in this setting.

Regarding the relationship between work schedule and turnover, the model including all the nurses of the 2018 NSSRN data showed that actual weekly work hours were positively related to nurse turnover (Table 2). Overtime hours per week had a non-linear relationship with nurse turnover (Table 2). In previous studies, voluntary overtime among newly licensed RNs was negatively related to nurse turnover (16). Among young nurses, an increase in overtime hours led to a decrease in turnover (7). The non-linear relationship in this study indicates that working 12 h or more per week is detrimental to nurse turnover. The negative relationship between 1 and 11 h of overtime and turnover can be interpreted differently. If paid, these overtime hours might be attractive for nurses to fulfill their financial needs (16). Furthermore, nurses who work overtime may provide greater help (24) and have high levels of organizational commitment (25), which lead to lower turnover. Although more studies should be conducted on this non-linear relationship of overtime and turnover, and find the appropriate levels of overtime work hours, the findings of this study suggest that 1–11 h of overtime per week might be suitable. Simultaneously, irrespective of mandatory or voluntary overtime, an increase in actual weekly work hours increased nurse turnover; therefore, nurses’ work hours and overtime should be monitored and regulated. In the U.S., several states regulate shift length and mandatory overtime for nurses (19). For example, the policy restricts working more than 12 consecutive work hours within a 24-h period, and nurses can refuse mandatory overtime requests from employers. This study supports such legislation and policy implementation and extension to reduce nurse turnover. For other countries without such policy, nurses’ associations and nurses need to advocate for the implementation of similar policy. Within healthcare organizations, nurses’ work hours and overtime should be managed according to this policy, and adherence to the policy should be monitored.

Another interesting finding is that the relationship between work schedule and nurse turnover differed across settings, being significant only in hospitals and other inpatient settings (Table 3). In clinic/ambulatory and other settings, this relationship was not significant (Table 4). Among nurses in hospitals, actual weekly work hours increased nurse turnover, while working 1–11 h of overtime per week decreased nurse turnover (Table 3). In contrast, for nurses working in other inpatient settings, overtime or weekly work hours increased turnover (Table 3). During the COVID-19 pandemic, nurses providing care to critical patients worked increased overtime hours which correlated with longer shift length (26). This study’s findings showed that to retain nurses in hospitals and inpatient settings it is important to ensure appropriate hours of work. Thus, staff nurses, nurse managers, and governments need to pay greater attention to nurses’ work schedules in these settings, as the pandemic persists.

In terms of other significant factors affecting nurse turnover, higher earnings from the primary nursing position decreased their turnover in the total model and the other three models, except for the other types of settings model (Tables 2–4). Higher job satisfaction decreased turnover in all models (Tables 2–4). Previous studies also found a negative relationship between salary and nurse turnover (7, 27). Job satisfaction is theoretically and empirically a significant contributing factor that decreases nurse turnover (28–30). This study confirmed these relationships. Further, monitoring and improving the salary and job satisfaction of nurses could be effective managerial strategies to reduce turnover. In the total model, as well as in clinic/ambulatory and other types of settings models, nurses working in non-unionized settings reported higher turnover than those working in unionized settings (Tables 2, 4). A previous study found this relationship among hospital nurses (29, 31). In total, 15.7% of the participants worked in unionized organizations (Table 1). Thus, according to this finding, unionization can be effective to reduce nurse turnover in public clinics or home health agency settings.

In each setting, different factors significantly affected nurses’ turnover. Among hospital nurses, the percentage of time spent in patient care significantly increased turnover (Table 3). In the 2018 NSSRN, the percentage of time for different nurse activities was collected, including patient care and charting, care coordination, management, research, teaching, non-nursing tasks, and others. When nurses spent a higher amount of time in patient care, they were more likely to leave their positions. During the COVID-19 pandemic, a greater number of nurses are needed at the bedside for patient care. Based on this study’s findings, to keep nurses at the bedside, it is important to monitor their patient care hours and ensure that they maintain an appropriate amount of patient load. In 2022, Minnesota enacted the Keeping Nurses at the Bedside Act to retain nurses and improve patient care, which included a nurse staffing policy (32). This study supports this policy change to reduce nurse turnover and improve patient care. Other countries should also consider developing such policy both at the organizational and national level to retain nurses during the pandemic.

Furthermore, this study found a negative relationship between age and turnover among hospital nurses, which showed young nurses leave the hospitals more (Table 3). Assuming that young nurses are newly licensed RNs, almost 90% of them began their nursing positions in a hospital setting (16). Their turnover rate is higher than that of experienced nurses (13). Through multivariate analysis, this study found that young age was associated with nurse turnover. Given that the average age of nurses was 47.73 years (Table 1), retaining young nurses, especially in a hospital setting, is a critical issue. Therefore, each hospital must undertake greater efforts to prevent young nurses from leaving. A resident program was found to be effective in retaining recent graduates (33). Government support for this program is essential. Other country experiencing high turnover among young nurses (7) also need to have government support to retain new graduates in hospitals.

In other inpatient settings, the effect of earnings on nurse turnover was relatively higher than in other settings (OR = 0.989, 95% CI = 0.982–0.996, Table 3). Sharma and Xu (21) examined the relationship between wages and turnover in nursing homes. It was found that high levels of wages were related to lower levels of turnover among certified nurse aides but not among licensed practical nurses and RNs. In this study, higher earnings from the primary position led to a lower turnover among nurses working in nursing homes, rehabilitation facilities, inpatient mental health, and inpatient hospices where the vulnerable population resided. Similarly, the effect of job satisfaction on nurse turnover was also higher than that in other settings (OR = 0.357, 95% CI = 0.299–427, Table 3). In a *post hoc* analysis, the level of earnings in this setting ranked third among the four settings. The level of job satisfaction was lowest, followed by hospital nurses. The COVID-19 pandemic has increased the vulnerability of the already vulnerable populations, including older individuals (21). Improvements in salary and job satisfaction of nurses working in this setting are needed to prevent turnover and ensure the provision of nursing care services for this population. Furthermore, many countries are presently experiencing a demographic change towards aging population, which emphasizes the importance of providing nursing care for older citizens. Using these strategies will help retain nurses in this setting.

The results of this study can be used as evidence to develop practice guidelines regarding nurse work scheduling in order to reduce nurse turnover. Specifically, longer weekly work hours and working more than 12 h overtime per week should be regulated. Simultaneously, nurse managers and nurses should understand state labor policies regarding nurse work hours and mandatory overtime, and adherence to such policies should be monitored. Nurse managers in different work settings can use specific factors contributing to turnover to develop retention strategies to avoid it. Regardless of work settings, salary and job satisfaction should be improved to reduce nurse turnover.

### Strengths and Limitations

A major strength of this study is its use of the nationally representative sample. However, this study had several limitations. The data of NSSRN were collected in 2018, meaning it is not recent. The data collection time varied across the participants, so the duration of the nurse turnover rate can be interpreted in several ways. It is difficult to estimate the annual turnover rates of participants. Further, nurse turnover reported by the nurses was not classified as voluntary or involuntary. In addition, several factors that might affect nurse turnover were not included in this study because of the absence of data (e.g., sleep, health status) (34, 35). This might have affected the study’s findings; therefore, they should be interpreted with caution. Work schedules were measured by actual weekly work hours and overtime hours per week. Future studies should include other components of the work schedule, such as shift length and breaks between and within shifts.

### Conclusion

Based on nationwide survey data in the United States, this study examined the relationship between nurses’ work schedule and turnover. A positive relationship was found between actual weekly work hours and nurse turnover and a non-linear relationship was found between overtime hours and turnover. Furthermore, the relationship between work schedules and turnover varied across different work settings. Additionally, nurses’ salary and job satisfaction were strong factors contributing to nurse turnover in all settings, with several factors (e.g., patient care in a hospital, unionized workplace in clinical/ambulatory, and other types of settings) significant in specific work settings. Nurse managers can use this study’s findings to develop retention strategies in their work settings. Considering the COVID-19 pandemic, nurse turnover has become a critical issue. Their work schedules, including work hours and overtime, should be monitored and regulated at the individual, institutional, and at state government levels.

Nurse managers must monitor and regulate staff nurses’ working hours and overtime, which can reduce the turnover. Particularly, nurses working overtime more than 12 h per week should be prohibited. Moreover, monitoring and regulating nurse work hours and overtime should be emphasized in hospitals and other inpatient settings. Currently, several states in the U.S. have implemented labor policies regarding nurses’ consecutive work hours and mandatory overtime. This study supports the implementation and expansion of the policy. Adherence to this labor policy should be evaluated in hospitals and other inpatient settings. Nurse managers and staff nurses need to advocate such policy changes. The findings of this study can be used to prevent nurse turnover across specific work settings.

## References

[1] Brook J Aitken L Webb R MacLaren J Salmon D. Characteristics of Successful Interventions to Reduce Turnover and Increase Retention of Early Career Nurses: A Systematic Review. Int J Nurs Stud (2019) 91:47–59. 10.1016/j.ijnurstu.2018.11.003 30669077

[2] Xu G Zeng X Wu X. Global Prevalence of Turnover Intention Among Intensive Care Nurses: A Meta‐analysis. Nurs Crit Care (2023) 28:159–66. 10.1111/nicc.12679 34261191

[3] Tang JH Hudson P. Evidence-based Practice Guideline: Nurse Retention for Nurse Managers. J Gerontol Nurs (2019) 45:11–9. 10.3928/00989134-20191011-03 31651984

[4] Bae SH. Noneconomic and Economic Impacts of Nurse Turnover in Hospitals: A Systematic Review. Int Nurs Rev (2022) 69:392–404. 10.1111/inr.12769 35654041 PMC9545246

[5] Li Y Jones CB. A Literature Review of Nursing Turnover Costs. J Nurs Manag (2013) 21:405–18. 10.1111/j.1365-2834.2012.01411.x 23406301

[6] Nelson-Brantley HV Park SH Bergquist-Beringer S. Characteristics of the Nursing Practice Environment Associated with Lower Unit-Level RN Turnover. J Nurs Adm (2018) 48:31–7. 10.1097/NNA.0000000000000567 29219908

[7] Bae SH Cho M Kim O Pang Y Cha C Jung H Predictors of Actual Turnover Among Nurses Working in Korean Hospitals: A Nationwide Longitudinal Survey Study. J Nurs Manag (2021) 29:2102–14. 10.1111/jonm.13347 33894028

[8] Ruiz PB Perroca MG Jericó Mde C. Cost of Nursing Turnover in a Teaching Hospital. Rev Esc Enferm USP (2016) 50:101–8. 10.1590/S0080-623420160000100014 27007427

[9] Back CY Hyun DS Jeung DY Chang SJ. Mediating Effects of Burnout in the Association between Emotional Labor and Turnover Intention in Korean Clinical Nurses. Saf Health Work (2020) 11:88–96. 10.1016/j.shaw.2020.01.002 32206378 PMC7078559

[10] O’Brien-Pallas L Murphy GT Shamian J Li X Hayes LJ. Impact and Determinants of Nurse Turnover: a Pan-Canadian Study. J Nurs Manag (2010) 18:1073–86. 10.1111/j.1365-2834.2010.01167.x 21073578

[11] Park SH Boyle DK Bergquist-Beringer S Staggs VS Dunton NE. Concurrent and Lagged Effects of Registered Nurse Turnover and Staffing on Unit-Acquired Pressure Ulcers. Health Serv Res (2014) 49:1205–25. 10.1111/1475-6773.12158 24476194 PMC4239846

[12] Winter V Schreyögg J Thiel A. Hospital Staff Shortages: Environmental and Organizational Determinants and Implications for Patient Satisfaction. Health Policy (2020) 124:380–8. 10.1016/j.healthpol.2020.01.001 31973906

[13] Kovner CT Djukic M Fatehi FK Fletcher J Jun J Brewer C Estimating and Preventing Hospital Internal Turnover of Newly Licensed Nurses: A Panel Survey. Int J Nurs Stud (2016) 60:251–62. 10.1016/j.ijnurstu.2016.05.003 27297385

[14] Halter M Boiko O Pelone F Beighton C Harris R Gale J The Determinants and Consequences of Adult Nursing Staff Turnover: A Systematic Review of Systematic Reviews. BMC Health Serv Res (2017) 17:824. 10.1186/s12913-017-2707-0 29246221 PMC5732502

[15] Stimpfel AW Sloane DM Aiken LH. The Longer the Shifts for Hospital Nurses, the Higher the Levels of Burnout and Patient Dissatisfaction. Health Aff (Millwood) (2012) 31:2501–9. 10.1377/hlthaff.2011.1377 23129681 PMC3608421

[16] Brewer CS Kovner CT Greene W Tukov-Shuser M Djukic M. Predictors of Actual Turnover in a National Sample of Newly Licensed Registered Nurses Employed in Hospitals. J Adv Nurs (2012) 68:521–38. 10.1111/j.1365-2648.2011.05753.x 22092452

[17] Bae SH. Relationships between Comprehensive Characteristics of Nurse Work Schedules and Adverse Patient Outcomes: A Systematic Literature Review. J Clin Nurs (2021) 30:2202–21. 10.1111/jocn.15728 33616252

[18] Min A Min H Hong HC. Work Schedule Characteristics and Fatigue Among Rotating Shift Nurses in Hospital Setting: An Integrative Review. J Nurs Manag (2019) 27:884–95. 10.1111/jonm.12756 30737987

[19] Bae SH Yoon J. Impact of States’ Nurse Work Hour Regulations on Overtime Practices and Work Hours Among Registered Nurses. Health Serv Res (2014) 49:1638–58. 10.1111/1475-6773.12179 24779701 PMC4213053

[20] Hayes LJ O’Brien-Pallas L Duffield C Shamian J Buchan J Hughes F Nurse Turnover: A Literature Review – an Update. Int J Nurs Stud (2012) 49:887–905. 10.1016/j.ijnurstu.2011.10.001 22019402

[21] Sharma H Xu L. Association between Wages and Nursing Staff Turnover in Iowa Nursing Homes. Innov Aging (2022) 6:igac004. 10.1093/geroni/igac004 35770065 PMC9233199

[22] US Department of Health and Human Services Health Resources and Services Administration National Center for Health Workforce Analysis. Brief Summary Results from the 2018 National Sample Survey of Registered Nurses (2019). Available at: https://bhw.hrsa.gov/sites/default/files/bureau-health-workforce/data-research/nssrn-summary-report.pdf (Accessed December 30, 2022).

[23] Faul F Erdfelder E Lang AG Buchner A. G*Power 3: a Flexible Statistical Power Analysis Program for the Social, Behavioral, and Biomedical Sciences. Behav Res Methods (2007) 39:175–91. 10.3758/bf03193146 17695343

[24] Bae SH Farasat A Nikolaev A Seo JY Foltz-Ramos K Fabry D Nursing Teams: Behind the Charts. J Nurs Manag (2017) 25:354–65. 10.1111/jonm.12473 28294446

[25] Bae SH Brewer CS Kovner CT. State Mandatory Overtime Regulations and Newly Licensed Nurses’ Mandatory and Voluntary Overtime and Total Work Hours. Nurs Outlook (2012) 60:60–71. 10.1016/j.outlook.2011.06.006 21872891

[26] Ren HF Chen FJ He LX Liu CQ Liu YY Huang YJ Nursing Allocation in Isolation Wards of COVID-19 Designated Hospitals: A Nationwide Study in China. BMC Nurs (2022) 21:23. 10.1186/s12912-021-00795-w 35042486 PMC8766220

[27] Kim S Lee K. Predictors of Turnover Among New Nurses Using Multilevel Survival Analysis. J Korean Acad Nurs (2016) 46:733–43. 10.4040/jkan.2016.46.5.733 27857016

[28] Brewer CS Chao YY Colder CR Kovner CT Chacko TP. A Structural Equation Model of Turnover for a Longitudinal Survey Among Early Career Registered Nurses. Int J Nurs Stud (2015) 52:1735–45. 10.1016/j.ijnurstu.2015.06.017 26212604

[29] Lee E. Why Newly Graduated Nurses in South Korea Leave Their First Job in a Short Time? A Survival Analysis. Hum Resour Health (2019) 17:61. 10.1186/s12960-019-0397-x 31358009 PMC6664533

[30] Price JL. Reflections on the Determinants of Voluntary Turnover. Int J Manpow (2001) 22:600–24. 10.1108/EUM0000000006233

[31] Cho SH Lee JY Mark BA Yun SC. Turnover of New Graduate Nurses in Their First Job Using Survival Analysis. J Nurs Scholarsh (2012) 44:63–70. 10.1111/j.1547-5069.2011.01428.x 22233430

[32] Minnesota Nurses Association. Keeping Nurses at the Bedside Act (2022). Available at: https://mnnurses.org/issues-advocacy/issues/top-legislative-issues/keeping-nurses-at-the-bedside-act/ (Accessed December 30, 2022).

[33] Van Camp J Chappy S. The Effectiveness of Nurse Residency Programs on Retention: A Systematic Review. AORN J (2017) 106:128–44. 10.1016/j.aorn.2017.06.003 28755665

[34] Han K Kim YH Lee HY Lim S. Pre-employment Health Lifestyle Profiles and Actual Turnover Among Newly Graduated Nurses: A Descriptive and Prospective Longitudinal Study. Int J Nurs Stud (2019) 98:1–8. 10.1016/j.ijnurstu.2019.05.014 31233956

[35] Han K Kim YH Lee HY Lim S. Novice Nurses’ Sleep Disturbance Trajectories within the First 2 Years of Work and Actual Turnover: A Prospective Longitudinal Study. Int J Nurs Stud (2020) 112:103575. 10.1016/j.ijnurstu.2020.103575 32404261

