# Supplementary material for: Association of Work Schedules With Nurse Turnover: A Cross-Sectional National Study
Source: Int J Public Health. 2023 Apr 24;68:1605732. doi: 10.3389/ijph.2023.1605732 (PMC10164927; doi:10.3389/ijph.2023.1605732)
Supplement: Supplementary file 1 [file Table1.docx]

Supplementary table

| Hospital (not mental health) | - Critical Access Hospital (CAH) – a rural community hospital that receives cost-based reimbursement   from Medicare   - Inpatient unit, not Critical Access Hospital - Emergency Department, not Critical Access Hospital - Hospital sponsored ambulatory care   (outpatient, surgery, clinic, urgent care, etc.)   - Hospital ancillary unit - Hospital nursing home unit - Hospital administration |
| --- | --- |
| Other inpatient setting | - Nursing home unit NOT in hospital - Rehabilitation facility/long-term care - Inpatient mental health/substance abuse - Correctional facility - Inpatient hospice |
| Clinic/Ambulatory | - Nurse managed health center - Private medical practice (clinic, physician   office, etc.)   - Public clinic (Rural Health Center, FQHC,   Indian Health Service, Tribal Clinic, etc.)   - School health service (K-12 or college) - Outpatient mental health/substance abuse - Urgent care (not hospital based) - Ambulatory surgery center (free standing) |
| Other types of settings | - Home health agency/service - Occupational health or employee health   service   - Public health or community health agency   (not a clinic)   - Government agency other than public/   community health or correctional facility   - Outpatient dialysis center - University or college academic department - Insurance company - Call center/telenursing center |
